# Prucalopride in the treatment of chronic constipation in patients from the Asia-Pacific region: a randomized, double-blind, placebo-controlled study

**DOI:** 10.1111/j.1365-2982.2012.01983.x

**Published:** 2012-10-11

**Authors:** M Ke, D Zou, Y Yuan, Y Li, L Lin, J Hao, X Hou, HJ Kim

**Affiliations:** * Clinical and Research Center of FGID & DGIM, Department of Gastroenterology, Peking Union Medical College HospitalBeijing, China; † Department of Gastroenterology, Changhai Hospital of ShanghaiShanghai, China; ‡ Department of Gastroenterology, Shanghai Ruijin HospitalShanghai, China; § Department of Gastroenterology, Qilu Hospital of Shangdong University affiliated to Capital Medical UniversityJinan, China; ¶ Department of Gastroenterology, Jiangsu Province HospitalNanjing, China; ** Department of Gastroenterology, Beijing Chaoyang HospitalBeijing, China; †† Department of Gastroenterology, Tongji Medical College, Union Hospital, Huazhong Science Technology UniversityWuhan, China; ‡‡ Department of Internal Medicine, Kyung Hee University HospitalSeoul, Korea

**Keywords:** Asian, Asia-Pacific, constipation, efficacy, prucalopride, safety

## Abstract

**Background:**

The study evaluated efficacy and safety of the 2 mg dose of prucalopride compared to placebo in patients with chronic constipation (CC) from the Asia-Pacific region.

**Methods:**

Randomized, placebo-controlled, parallel-group, phase III study with 2-week run-in, 12-week treatment phase, and 1-week follow-up. Adult patients with CC (≤2 spontaneous bowel movements per week) received 2 mg prucalopride or placebo, once-daily, for 12 weeks. Primary efficacy measure was percentage of patients with average of ≥3 spontaneous complete bowel movements (SCBMs) per week (Responders) during the 12-week treatment. A key secondary endpoint was Responders during first 4 weeks of treatment. Other efficacy assessments were based on patient diaries, their assessments of symptoms and quality of life, and investigator’s assessment on efficacy of treatment. Safety assessments included adverse events, laboratory values, and cardiovascular events.

**Key Results:**

Efficacy and safety were evaluated for 501 patients who received study drug. On the primary endpoint, prucalopride was significantly more effective than placebo with 83 (33.3%) *vs* 26 (10.3%) patients having a weekly average of ≥3 SCBMs during the 12-week treatment (*P* < 0.001). Respective percentages were 34.5%*vs* 11.1% over first 4 weeks (*P* < 0.001). On other secondary endpoints, clinical improvement was generally larger and statistically superior (*P* < 0.001) in the prucalopride group. Most frequently reported adverse events were diarrhea, nausea, abdominal pain, and headache.

**Conclusion & Inferences:**

Prucalopride 2 mg given once-daily significantly improved bowel function, associated symptoms, and satisfaction in CC over a 12-week treatment period, and was safe and well tolerated by patients in the Asia-Pacific region.

## Introduction

Constipation is a common digestive complaint and a collective term used by the patient to imply that stools are too hard, too infrequent, or too difficult to pass. Constipation can be a debilitating medical problem and a symptom of many diseases.^1^^,^^2^ The prevalence of chronic constipation (CC) is probably underestimated as many patients may not seek medical advice for the condition, but its impact on quality of life and economic burden has been demonstrated.^3–5^ Constipation is more common in women and elderly.^6–9^

Prucalopride, the first representative of a novel chemical class (benzofurans) of dihydrobenzofurancarboxamide derivatives, induces giant migrating contractions of the bowel, stimulates proximal colonic motility, enhances gastro-pyloro-duodenal motility, and accelerates gastric emptying by specific and selective stimulation of serotonin 5-hydroxytryptamine 4 (5-HT_4_) receptors.^10^ Stimulation of the 5-HT_4_ receptor induces facilitation of cholinergic and non-cholinergic excitatory neurotransmission, and hence prucalopride has potential for the treatment of disorders associated with small and/or large bowel dysfunction, including constipation, postoperative ileus, and pseudo-obstruction.^11^^,^^12^

The safety and efficacy of prucalopride in CC has been investigated in an extensive development program,^10^^,^^13–17^ which included three pivotal clinical studies.^18–20^ In these randomized, double-blind, placebo-controlled studies, adults (18–95 years) with CC received 2 or 4 mg prucalopride tablets once-daily for 12 weeks. Across studies, both doses of prucalopride were statistically superior to placebo on the primary endpoint defined as an average of 3 or more spontaneous, complete bowel movements (SCBMs) per week over the treatment period. There were also significant benefits of prucalopride on other measures, including patient-reported outcome measures (e.g., satisfaction with treatment and with bowel movements, physical and psychosocial discomfort). There were no major safety issues, and assessment of long-term safety data did not reveal any new emerging safety signals.^21^

Prucalopride has been approved in 27 European Union countries and other countries or region (e.g., Iceland, Liechtenstein Norway, Switzerland, Aruba, Canada, Chile, Honduras, Hong Kong, Jamaica, Macau, Malaysia, Panama, Peru, Philippines, Russia, and Syria) for the treatment of women with CC in whom laxatives fail to provide adequate relief; in Colombia, Mexico, New Zealand, and Singapore, the approval also includes men. Prucalopride has also been approved in Australia for the treatment of chronic functional constipation in adults in whom laxatives fail to provide adequate relief. The recommended dose of the drug in adults in most countries is 2 mg daily.^22^^,^^23^

Consistent with the observations from Western populations, functional constipation in China and other Asian populations has been reported as more prevalent in women and in older people and as having negative effects on quality of life.^9^^,^^24^ Previous clinical studies of prucalopride were conducted predominantly in adult patients of Caucasian origin, with no published studies to date in the Asian population. Ethnicity has the potential to affect a drug’s efficacy and safety profile, possibly as a result of pharmacokinetic and pharmacologic variations.^25–27^ Therefore, this clinical study was conducted to evaluate and confirm the efficacy and tolerability of prucalopride in patients with CC in the Asia-Pacific region.

This study was modeled after the previous three pivotal studies conducted primarily in adult patients of Caucasian origin,^18–20^ and adults with CC from the Asia-Pacific region received prucalopride 2 mg or placebo orally once-daily for 12 weeks.

## Materials and Methods

### Study design

This randomized, double-blind, placebo-controlled, parallel-group, multicenter, phase 3 study was conducted at 46 sites of five countries/regions from April 2010 to March 2011. The study consisted of a 2-week drug-free screening/run-in phase, a 12-week, double-blind, placebo-controlled treatment phase, and a post-treatment follow-up 7 days following last dose of study drug.

The purpose of the screening/run-in phase (Visit 1) was to confirm patient’s eligibility for the study. The investigator completed screening assessments. Patients were instructed to stop all laxative intakes, not to change their diet or lifestyle, and given a diary to note the date and time of their bowel movements (BMs) and record the consistency of each BM using the Bristol Stool Form Scale (BSFC), the need to strain while defecating, and the sensation of complete evacuation. If a colonoscopy was performed at Visit 1, patients waited 1–4 weeks before starting the daily diary, and at least 3 weeks elapsed between Visits 1 and 2 for these patients. If the definition of constipation was not met during the run-in phase, the patient was ineligible for the treatment phase.

The double-blind treatment phase consisted of five visits every 2–4 weeks (Weeks 0, 2, 4, 8, and 12) over a 12-week period. Patients took the first dose of study drug on the first day after Visit 2 and then took one tablet daily before breakfast during the treatment period.

Patients recorded study drug and rescue medication dosing information and information related to bowel movements in a daily diary throughout the study. Patients completed efficacy assessments and questionnaires at specified visits and the investigator provided a global assessment of efficacy of treatment. Safety was monitored throughout the study. The end-of-treatment/early withdrawal visit was performed at Week 12 or at the time a patient withdraws from the study. A telephone post-treatment follow-up contact (or optional study visit, at the discretion of the investigator) was conducted for all patients approximately 7 days after the last dose of study drug to complete the evaluations.

The study was approved by the Independent Ethics Committee or Institutional Review Board and was conducted in accordance with the ethical principles that have their origin in the Declaration of Helsinki, consistent with Good Clinical Practices, and applicable regulatory requirements. All the patients provided written informed consent before entering the study.

### Study participants

Men and women aged 18–65 years with a history of CC were eligible for the study. A history of CC for ≥6 months before the screening visit was required to enter the run-in phase, and those who met the criteria during the 2-week run-in were eligible for randomization. History of CC was defined as follows: (i) ≤2 spontaneous BMs (SBMs) per week on average, and (ii) ≥1 of the following in >25% of BMs: very hard and/or hard stools, sensation of incomplete evacuation, straining at defecation, sensation of ano-rectal obstruction or blockade, or a need for digital manipulation to facilitate evacuation. These criteria were not met if SBM was preceded within 24 h by the intake of a laxative agent or by the use of an enema. Patients who never had SBMs were considered to be constipated. Constipation needed to be functional with no secondary causes of CC.

Exclusion criteria included the following: drug-induced constipation, patients suffering from secondary causes of chronic constipation, including endocrine, metabolic, or neurological disorders, surgical obstruction, megacolon/megarectum, or diagnosis of pseudo-obstruction. Other exclusion criteria were uncontrolled cardiovascular, liver and lung diseases, impaired renal function (serum creatinine >180 *μ*mol L^−1^), and clinically significant abnormal laboratory values.

### Study drug and rescue medication

The trial medication, prucalopride or matching placebo, was provided by the sponsor. The film-coated tablets for oral administration contained prucalopride succinate equivalent to 2 mg prucalopride base. During the 12-week treatment phase, prucalopride tablets or matching placebo were taken orally in the morning before breakfast or in the morning if no breakfast. Bisacodyl (5, 10, or 15 mg) was allowed as a rescue medication if the patient did not have a BM for ≥3 consecutive days. If the investigator decided to prescribe bisacodyl, and the dose was insufficient, an increase in dose was allowed up to a maximum single dose of 15 mg day^−1^. If no BMs were passed after an increase in the amount of bisacodyl, an enema could have been administered. No bisacodyl was to be taken or enemas used within 48 h before or after the first dose of study drug. Patients recorded study drug and rescue medication usage in a daily diary throughout the study.

### Efficacy evaluations

Efficacy evaluations were based on information recorded in the patient daily diary and their global evaluation on changes of consistency of stool, severity of constipation, and efficacy of treatment, and the investigator’s global assessment on efficacy of treatment.

Patients kept a daily diary during the study and recorded the following information: date and time of the intake of study drug; date and time a BM was produced and consistency of the stool, degree of straining, feeling of complete evacuation after a BM was passed; and date and time of the intake of bisacodyl and number of tablets, or use of enema.

The patient recorded the severity of symptoms occurring during the 2 weeks preceding the visit using the Patient Assessment of Constipation-Symptom questionnaire (PAC-SYM) and Patient Assessment of Constipation-Quality of Life questionnaire (PAC-QOL).^28^^,^^29^ The PAC-SYM contains three subscales: stool symptoms (five items), abdominal symptoms (four items), and rectal symptoms (three items). The PAC-QOL is a self-administered questionnaire for patients with constipation. The PAC-QOL contains 28 items within four subscales: physical discomfort (four items), psychosocial discomfort (eight items), worries and concerns (11 items), and satisfaction (five items). Changes (deteriorations or improvements) in symptoms captured by the PAC-SYM or PAC-QOL were not considered adverse events unless determined to be so by the investigator.

The investigator reviewed the patient daily diary and global evaluation on changes of consistency of stool, severity of constipation, and efficacy of treatment and then completed the global evaluation.

### Efficacy endpoints

The primary efficacy endpoint was the percentage of patients with an average of 3 or more SCBMs per week (Responders) during the entire 12-week double-blind treatment phase. The key secondary endpoint was the percentage of responders during the first 4 weeks of the double-blind treatment phase. Other secondary efficacy endpoints include percentage of patients with an average increase of ≥1 SCBM per week over 12-week study treatment period, percentages and averages change of bowel movements based on data collected on the diaries, time (days) to first SCBM after first intake of trial medication, changes from baseline in patient’s global assessments and PAC-SYM and PAC-QOL, and the investigator’s global assessment on efficacy of treatment.

### Safety assessments

Treatment-emergent adverse events were monitored throughout the study. Physical examinations, 12-lead ECG recordings, and routine laboratory tests (hematology/biochemistry/urinalysis) were performed at Visits 1, 4, and 6, or at early withdrawal. Vital signs and body weight were measured at each visit.

## Statistical Methods

### Sample size

Sample size estimation was based on the assumption that between-treatment difference in the primary endpoint was 12.3% (with 15% responders from the placebo group and 27.3% from the 2 mg group in the Asian population). A sample size of 237 patients per group was required to detect this difference with approximately 90% power (for a 2-sided test at 5% significance level). If approximately 5% of patients had insufficient diary data to be evaluated for the intent-to-treat (ITT) analysis set, 250 patients randomized to each treatment groups would be sufficient. The ITT analysis set included all randomized patients who received at least one dose of study drug and was used for efficacy and safety data analyses.

### Randomization

Patients were randomly assigned in a 1 : 1 ratio to 1 of 2 treatment groups (prucalopride or placebo) based on a computer-generated randomization schedule generated by the sponsor before the study. Randomization was balanced by using permuted blocks and stratified by investigator/country and run-in status of severity of constipation (i.e., <1 SBM and ≥1 and ≤2 SBM per week at baseline). The block size in randomization was unknown to the investigator sites and study team. Study drug was packaged and labeled based on the randomization schedule and treatment code. Treatment code for the patient was kept blind to the investigator. To maintain study blind, the study drug container had a multipart label and study drug information was not included.

### Efficacy analysis

The primary endpoint was the percentage of patients with an average of 3 or more SCBMs per week (responders) during the entire 12-week treatment phase. A Cochran-Mantel-Haenszel chi-squared test for general association between the treatment and response during the treatment phase was performed controlling for effects of investigator/country and baseline severity of constipation. The baseline severity of constipation was defined as ‘more severe’ if the patient had <1 SBM per week and as ‘less severe’ if the patient had ≥1 and ≤2 SBMs per week at baseline. The between-treatment difference in percent of responders and the 95% confidence interval (CI) of the difference were estimated. Similar statistical analysis methods were applied in the analysis of the key secondary endpoint (responders during first 4 weeks of treatment) and other dichotomous variables, such as the number (%) of patients with average increase of ≥1 SCBM per week, number (%) of patients rating their treatment as extremely or quite a bit effective, and other rates.

For continuous variables, an analysis of covariance (ancova) model with treatment, baseline severity of constipation, and investigator/country as factors and the baseline value as the covariate was used to assess the treatment effect as measured by the changes from baseline in the variable. The Van Elteren test controlling for investigator/country and baseline severity of constipation was used to assess the effect of treatment for the ordinal categorical variables. Time to first SCBM after first intake of trial medication was analyzed with the methods for survival data; Kaplan–Meier estimates were used to describe the distribution and the Log-rank test was performed for the between-treatment group comparison.

Odds ratio and the 95% CI for the placebo and prucalopride groups were estimated to assess the association between the response and several assessments: subjects’ evaluation of treatment as effective (‘quite a bit’ or ‘extremely’ effective), improvement ≥1 on PAC-SYM, and improvement ≥1 on PAC-QOL Overall Score.

### Safety analyses

Safety was evaluated by examining the incidence and types of adverse events, and changes in clinical laboratory test values, physical examination results, 12-lead ECGs, and vital sign measurements from the screening phase through study completion. Baseline was the last evaluation performed before study drug administration. Descriptive statistics of corrected QT (QTc) intervals and changes from baseline were summarized at each scheduled time point to detect individual QTc changes.

## Results

### Patient characteristics

**Figure 1:**
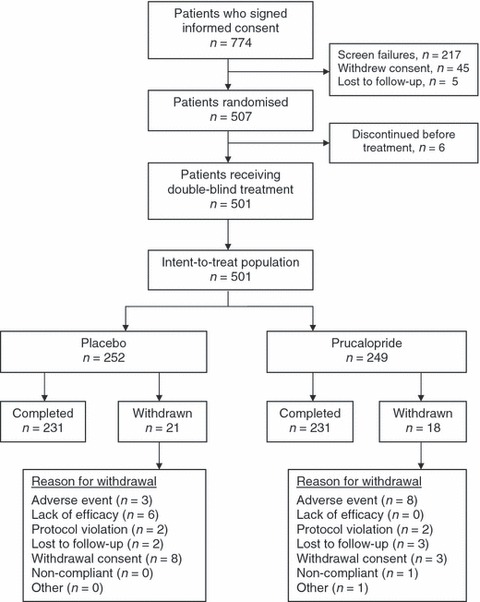
CONSORT diagram showing the flow of participants through each stage of the trial. The number of patients in each stage or treatment arm is included.

A total of 774 patients were screened at 46 sites in five countries/regions, and 501 patients randomized to placebo or prucalopride treatment arms took at least one dose of study drug and were included in the ITT population (Fig. 1). A total of 462 patients (92.2%) completed the double-blind treatment phase with a discontinuation rate of 7.8%. Approximately 92% of patients in each treatment group were treatment compliant, and exposure to study drug was similar for the placebo and prucalopride groups with an average of 79.5 and 79.6 days on drug, respectively.

Patient demography and constipation history are provided for all treated patients in Table 1. The majority was Asian (92.4%), women (89.8%), and average patient’s age and weight were 41.6 years and 59.2 kg, respectively. The mean duration of constipation was 12.9 years and 22.8% of patients reported no spontaneous BMs during the 6 months before study entry. Approximately three quarters of patients reported prior laxative and/or enema use within the 6 months preceding study entry, with only 23.1% (83/360) of these patients reporting that use of these therapies had been ‘adequate’. Patient characteristics including constipation history were well matched between the two treatment groups.

**Table 1:** Demographic data and constipation history in all treated patients

Characteristics	Placebo (*N *= 252)	Prucalopride (*N* = 249)	Overall (*N* = 501)
Race, *n* (%)			
Asian	231 (91.7)	232 (93.2)	463 (92.4)
White	19 (7.5)	12 (4.8)	31 (6.2)
Other	2 (0.8)	5 (2.0)	7 (1.4)
Gender, *n* (%)			
Female	223 (88.5)	227 (91.2)	450 (89.8)
Male	29 (11.5)	22 (8.8)	51 (10.2)
Age, years			
Mean (SD)	41.8 (12.88)	41.4 (12.92)	41.6 (12.89)
Range (min–max)	(18;65)	(18;65)	(18;65)
Height, cm			
Mean (SD)	162.7 (7.30)	161.7 (6.36)	162.2 (6.86)
Range (min–max)	(145;192)	(147;179)	(145;192)
Weight, kg			
Mean (SD)	59.1 (10.29)	59.2 (10.00)	59.2 (10.14)
Range (min–max)	(38;93)	(40;106)	(38;106)
Reported duration of constipation, years			
Mean (SD)	12.8 (9.97)	12.9 (9.75)	12.9 (9.85)
Range (min–max)	(0.5;45.5)	(0.7;60.0)	(0.5;60.0)
Reported average frequency of spontaneous stools per week, *n* (%)			
No spontaneous stools	57 (22.6)	57 (22.9)	114 (22.8)
>0 and ≤1	63 (25.0)	73 (29.3)	136 (27.1)
>1 or ≤2	132 (52.4)	119 (47.8)	251 (50.1)
Overall assessment of therapeutic effect of previous treatment for constipation, *n* (%)			
Not used	75 (29.8)	66 (26.5)	141 (28.1)
Used and adequate	40 (15.9)	43 (17.3)	83 (16.6)
Used and inadequate	137 (54.4)	140 (56.2)	277 (55.3)

### Primary efficacy endpoint

**Figure 2:**
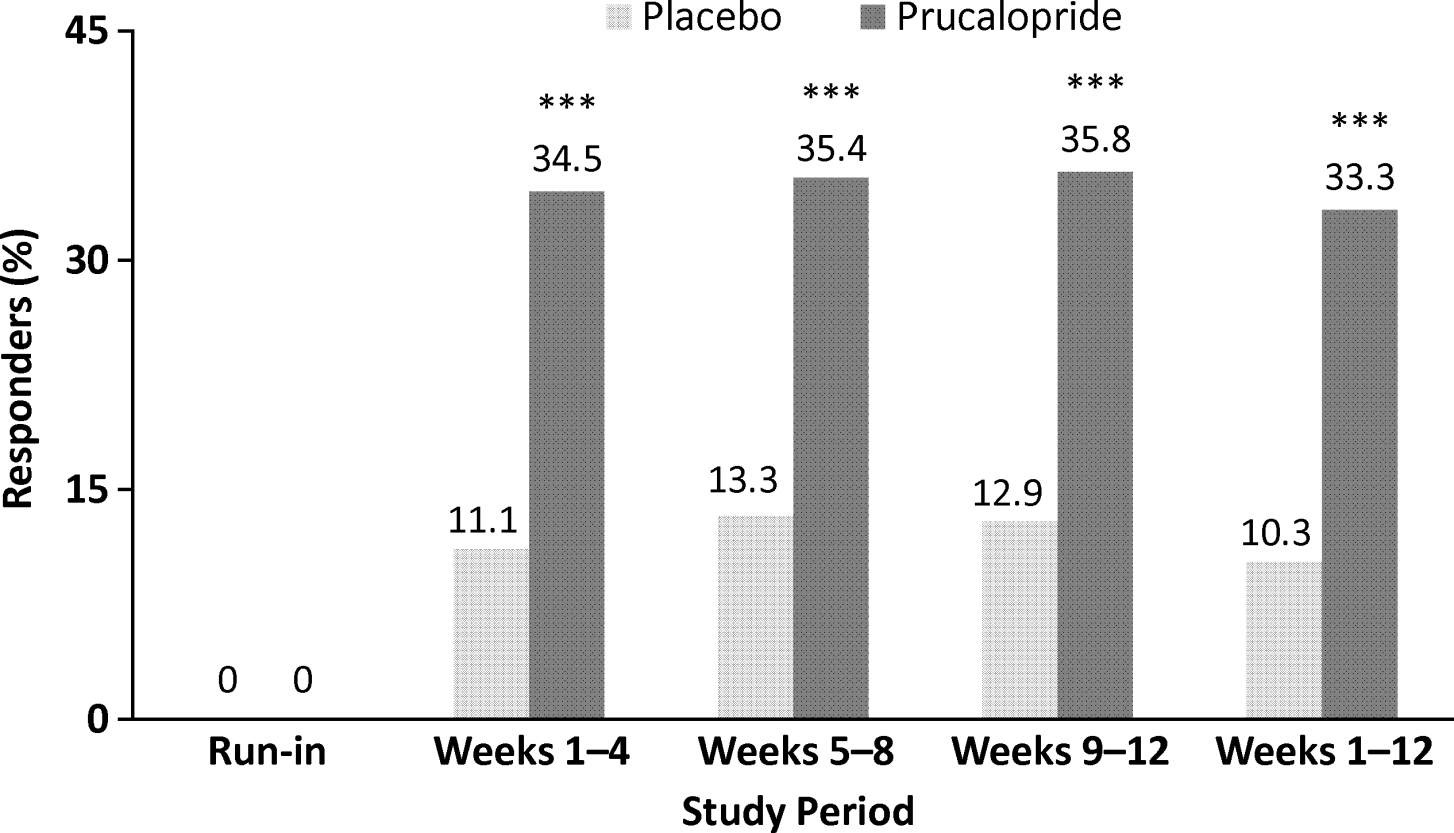
Percentage of patients in each treatment group with an average of 3 or more spontaneous complete bowel movements per week (Responders). Data were averaged over every 4 weeks (weeks 1–4, 5–8, 9–12) or weeks 1–12. ***Denote significant differences *vs* placebo (*P* < 0.001).

During the run-in phase (baseline), patients reported an average of 1.1 SBMs per week and an average of 0.3 SCBM per week. Prucalopride treatment compared with placebo resulted in a significantly higher (*P* < 0.001) percentage of patients with an average of 3 or more SCBMs per week during the 12-week treatment phase (Table 2, Fig. 2). These percentages were 33.3% for prucalopride *vs* 10.3% for placebo, representing a therapeutic gain of 23.0% (95% CI = 16.1–30.0%; *P* < 0.001) with prucalopride over placebo. Over 12 weeks, the therapeutic gain with prucalopride treatment compared with placebo was consistently higher (*P*≤ 0.031) regardless of patients’ age, gender, race, or use of prior therapy (laxative/enema).

**Table 2:** Percentage of patients with an average of ≥3 SCBMS per week during weeks 1–12

	Placebo(*N *= 252)	Prucalopride(*N *= 249)	*P* value*
Primary efficacy endpoint			
Responders, *n* (%)	26 (10.3)	83 (33.3)	<0.001
Subgroups, *n*/*N* (%)			
Race			
Asian	25/231 (10.8)	78/232 (33.6)	<0.001
Non-Asian	1/21 (4.8)	5/17 (29.4)	0.031
Gender			
Female	24/223 (10.8)	77/227 (33.9)	<0.001
Male	2/29 (6.9)	6/22 (27.3)	0.022
Age group			
18–40 years	11/107 (10.3)	41/115 (35.7)	<0.001
41–65 years	15/145 (10.3)	42/134 (31.3)	<0.001
Previous treatment for constipation			
Not used	13/75 (17.3)	33/66 (50.0)	<0.001
Used and adequate	1/40 (2.5)	15/43 (34.9)	<0.001
Used and inadequate	12/137 (8.8)	35/140 (25.0)	<0.001

SCBM, spontaneous complete bowel movement.

* Levels of significance: prucalopride *vs* placebo.

### Key secondary efficacy endpoint

Over the first 4 weeks, an average of 3 or more SCBM per week was achieved by 34.5% of patients treated with prucalopride *vs* 11.1% on placebo, representing a therapeutic gain of 23.4% (95% CI = 16.4–30.5%; *P* < 0.001) with prucalopride (Fig. 2).

### Other efficacy endpoints

The proportion of patients with 3 or more SCBM per week was also significantly greater (*P* < 0.001) with prucalopride than placebo during weeks 5–8 and weeks 9–12 (Fig. 2).

Secondary efficacy parameters from diaries and patient global assessment questionnaires were significantly (*P* < 0.001) improved with prucalopride compared with placebo (Table 3). During the 12-week treatment period, patients treated with prucalopride showed significantly greater (*P* < 0.001) average increase of ≥1 SCBM per week and shorter time to first SCBM after first intake of trial medication, along with improved patient assessment of constipation as absent or mild and rating of treatment as extremely or quite a bit effective. Prucalopride also showed significantly greater improvements (*P* < 0.001) in the mean change from baseline on average number of SCBM per week, consistency per BM, straining per BM, and reduction in number of days with laxative use or enema per week, and bisacodyl tablets taken per week.

**Table 3:** Other efficacy data from diaries and patient global assessment questionnaires

Assessment	Placebo (*N *= 252)	Prucalopride (*N *= 249)	*P* value*
Patients with an average increase of ≥1 SCBM per week, *n*/*N* (%)			
Weeks 1–12	68/248 (27.4)	139/243 (57.2)	<0.001
Average number of SCBM per week, mean (mean change from baseline)			
Baseline	0.3	0.3	<0.001
Weeks 1–12	1.1 (0.8)	2.4 (2.1)	
Average consistency per BM, mean (mean change from baseline)			
Baseline	3.4	3.4	<0.001
Weeks 1–12	3.6 (0.1)	4.0 (0.7)	
Average straining per BM, mean (mean change from baseline)			
Baseline	1.9	2.0	<0.001
Weeks 1–12	1.7 (−0.2)	1.3 (−0.7)	
Time to first SCBM after first intake of trial medication, days			
Median time (range)	12.58 (9.02; 20.09)	1.56 (1.01; 3.06)	<0.001
Average number of days with laxative or enema use/week, mean (mean change from baseline)			
Baseline	0.9	1.0	<0.001
Weeks 1–12	0.7 (−0.2)	0.3 (−0.6)	
Average bisacodyl tablets taken/week, mean (mean change from baseline)			
Baseline	1.6	1.7	<0.001
Weeks 1–12	1.3 (−0.3)	0.6 (−1.0)	
Patient assessment of constipation as absent or mild, *n*/*N* (%)†			
Baseline	12/252 (4.8)	8/249 (3.2)	<0.001
Week 12 LOCF	68/249 (27.3)	133/249 (53.4)	
Patients rating their treatment as extremely or quite a bit effective, *n*/*N* (%)			
Week 12 LOCF	22/249 (8.8)	82/249 (32.9)	<0.001
Investigator evaluating the treatment as extremely or quite a bit effective, *n*/*N* (%)			
Week 12 LOCF	34/247 (13.8)	101/247 (40.9)	<0.001

BM, bowel movement; LOCF, last observation carried forward; SCBM, spontaneous complete bowel movement.

* Levels of significance: prucalopride *vs* placebo.

† At baseline, absent was not reported by any patient in placebo or prucalopride; at Weeks 1–12, absent reported by 19/249 patients in placebo and 62/249 patients in prucalopride.

The results of the PAC-SYM Questionnaires at baseline and at Week 12 last observation carried forward (LOCF) are summarized in Table 4. A reduction from baseline indicates an improvement in the PAC-SYM score. The mean reductions from baseline in the PAC-SYM overall score, and scores for stool, abdominal, and rectal symptoms, were significantly (*P* < 0.001) greater in the prucalopride group than in the placebo group at Week 12 (LOCF).

**Table 4:** Efficacy data derived from the PAC-SYM questionnaire

Assessment†	Placebo (*N *= 252)	Prucalopride (*N *= 249)	*P* value*
Overall PAC-SYM symptoms score, mean (mean change from baseline)			
Baseline	1.5	1.5	<0.001
Week 12 LOCF	1.2 (−0.4)	0.8 (−0.7)	
PAC-SYM stool symptoms score, mean (mean change from baseline)			
Baseline	2.2	2.1	<0.001
Week 12 LOCF	1.7 (−0.5)	1.2 (−1.0)	
PAC-SYM abdominal symptoms score, mean (mean change from baseline)			
Baseline	1.1	1.2	<0.001
Week 12 LOCF	0.9 (−0.3)	0.6 (−0.6)	
PAC-SYM rectal symptoms score, mean (mean change from baseline)			
Baseline	0.9	1.0	<0.001
Week 12 LOCF	0.7 (−0.3)	0.4 (−0.5)	

LOCF, last observation carried forward.

* Levels of significance: prucalopride *vs* placebo.

† Decreases in score reflect improvement. Symptoms are rated on a 5-point scale: 0 (absent) to 4 (very severe).

The results of the PAC-QOL questionnaires at baseline and at Week 12 (LOCF) are summarized in Table 5. The mean reductions from baseline in the PAC-QOL overall score, and in the subscale scores for dissatisfaction, physical discomfort, psychosocial discomfort, and worries and concerns, were significantly greater (*P* < 0.001) in the prucalopride group than in the placebo group at Week 12 (LOCF).

**Table 5:** Quality of Life endpoints derived from PAC-QOL questionnaire

Assessment†	Placebo (*N *= 252)	Prucalopride(*N *= 249)	*P* value*
Overall PAC-QOL score, mean (mean change from baseline)			
Baseline	1.9	1.8	<0.001
Week 12 LOCF	1.5 (−0.4)	1.1 (−0.8)	
PAC-QOL dissatisfaction subscale score, mean (mean change from baseline)			
Baseline	3.0	3.0	<0.001
Week 12 LOCF	2.8 (−0.2)	2.1 (−0.9)	
PAC-QOL physical discomfort subscale score, mean (mean change from baseline)			
Baseline	1.7	1.7	<0.001
Week 12 LOCF	1.3 (−0.4)	0.9 (−0.8)	
PAC-QOL psychosocial discomfort subscale score, mean (mean change from baseline)			
Baseline	1.3	1.2	<0.001
Week 12 LOCF	0.9 (−0.4)	0.6 (−0.6)	
PAC-QOL worries and concerns subscale score, mean (mean change from baseline)			
Baseline	2.0	1.8	<0.001
Week 12 LOCF	1.5 (−0.5)	1.1 (−0.8)	

LOCF, last observation carried forward.

* Levels of significance: prucalopride *vs* placebo.

† Decreases in score reflect improvement.

Results from the analyses of association between responders (SCBM ≥3 per week, at Weeks 1–12) on selected assessments are summarized in Table 6. The results in both placebo and prucalopride 2 mg groups indicated that the responses to the treatments were significantly associated with subject evaluation of treatment effects as ‘quite a bit’ or ‘extremely’ effective (estimated odds ratios were >1 and 95% CIs excluded the value 1 for both treatment groups). The results in both placebo and prucalopride 2 mg groups indicated that the efficacy response to the treatments was significantly associated with the improvement ≥1 (Yes or No) in the PAC-SYM overall score (with odds ratios >1 and 95% CI excluded 1 in both treatment groups). Similarly, there were significant associations between the efficacy response and improvement in PAC-QOL overall score in both treatment groups.

**Table 6:** Analysis of association between responders (SCBM ≥3 per week, at Week 1–12) on selected assessments (Week 12 LOCF)

	Placebo (*N *= 252)			Prucalopride (*N *= 249)				
Assessment	Responder	Non-responder	Responder	Non-responder
Subject evaluation of treatment as ‘quite a bit’ or ‘extremely’ effective				
Yes	13	9	51	31
No	13	214	32	135
All	26	223	83	166
Odds ratio (95% CI)	23.78 (8.59, 65.79)			6.94 (3.85, 12.52)				
Improved ≥1 on PAC-SYM overall score				
Yes	9	33	38	48
No	17	190	45	118
All	26	223	83	166
Odds ratio (95% CI)	3.05 (1.25, 7.41)			2.08 (1.20, 3.59)				
Improved ≥1 on PAC-QOL overall score				
Yes	11	30	42	50
No	15	191	41	115
All	26	221	83	165
Odds ratio (95% CI)	4.67 (1.96, 11.12)			2.36 (1.37, 4.06)				

LOCF, last observation carried forward.

Subjects who were responders and rated the study as effective (or improvement ≥1 on PAC-SYM/QOL scores), and non-responders who rated the study as not effective (or improvement <1 on PAC-SYM/QOL scores) in the placebo or prucalopride groups showed perfect associations in these two measurements. However, some subjects reported treatment satisfaction or QOL improvements, but were not ‘responders’ based on the definition of SCBM ≥3 week^−1^ (at Week 1–12), and there were some subjects who were responders but did not meet the treatment satisfaction and QOL thresholds, which suggested that the improvement in number of SCBMs was associated with a lesser degree of perceived treatment satisfaction and QOL improvement or the other way around.

### Safety

A summary of treatment-emergent adverse events is provided in Table 7. The percentage of patients who reported at least one adverse event was higher in the prucalopride than the placebo group. Five patients in the placebo group and three patients in the prucalopride group experienced a serious adverse event during the study. A small number of patients had adverse events that led to study discontinuation in the prucalopride and placebo groups. Adverse events considered by the investigator to be drug related (very likely, probably, possibly, and events with missing relationship) occurred in 33 patients in the placebo group and 90 patients in the prucalopride group.

**Table 7:** Summary of treatment-emergent adverse events (all treated patients)

Adverse event, *n* (%)†	Placebo (*N *= 252)	Prucalopride (*N *= 249)
Any adverse event	92 (36.5)	142 (57.0)
Serious adverse event	5 (2.0)	3 (1.2)
Discontinuation due to adverse event	3 (1.2)	8 (3.2)
Study drug related adverse event*	33 (13.1)	90 (36.1)
Deaths	0	0
Adverse event ≥5% in prucalopride group		
Diarrhea	20 (7.9)	55 (22.1)
Headache	5 (2.0)	31 (12.4)
Nausea	8 (3.2)	29 (11.6)
Abdominal pain	6 (2.4)	17 (6.8)
Prespecified adverse events of interest		
Palpitations	3 (1.2)	4 (1.6)
ECG signs of myocardial ischemia	0	1 (0.4)
Pregnancy-associated events	1 (0.4)	0

* Includes relationship of ‘possibly’, ‘probably’, ‘very likely’, and events with missing relationship.

† Incidence is based on the number of patients experiencing at least one adverse event, not the number of events.

The most frequently reported adverse events in the prucalopride group were diarrhea, headache, nausea, and abdominal pain, and each was reported more often than in the placebo group. The occurrence of prespecified treatment-emergent adverse events of interest (palpitations, cardiovascular ischemic events, QT prolongation, cardiac arrhythmias, and pregnancy-associated events) occurred at a similar low rate in the prucalopride and placebo groups.

In the prucalopride group, only one of the serious adverse events was considered possibly related to study drug (ECG signs of myocardial ischemia). In the placebo group, one serious event (intrauterine death) was assessed as possibly related to study drug and another (erysipelas) was assessed as doubtfully related. All serious events had resolved or were resolving, except for the event of intrauterine death in the placebo group which occurred after the completion of study. No deaths were reported during this study.

In the prucalopride group, the most common adverse event leading to study drug withdrawal was diarrhea. The adverse events leading to discontinuation in the prucalopride group resolved, with the exception of lichen planus which was ongoing as of final follow-up. In each treatment group, one serious adverse event led to study drug discontinuation (ECG signs of myocardial ischemia in prucalopride; dizziness in placebo).

The findings for hematology and blood chemistry analytes, pulse rate, blood pressure, and ECG parameters were generally similar in the placebo and prucalopride groups. Among patients in the placebo group with normal baseline values, four patients had an abnormally low postbaseline heart rate and three had an abnormally elevated PR interval postbaseline. Patients with prolonged QTcB and QTcF intervals were similar in the placebo and prucalopride groups. Few patients in either treatment group had a prolonged QTcB or QTcF classification value at Week 4 or Week 12. No patient receiving prucalopride had a normal baseline value and an abnormal value on any postbaseline heart rate, PR interval, QRS interval, or QT interval (see Supporting Information).

## Discussion

This double-blind, randomized, placebo-controlled study evaluated the efficacy and safety of the 2 mg dose of prucalopride administered once-daily in patients with CC from the Asia-Pacific region. The therapeutic benefit of prucalopride *vs* placebo was demonstrated on the primary and secondary measures of efficacy. A significantly higher percentage of patients receiving prucalopride achieved normalization of BMs, defined as an average of 3 or more SCBMs per week, over the 12-week treatment period. This clinically meaningful treatment effect was consistently seen regardless of patient’s gender (male, female), age (18–40 and 41–65 years), race (Asian, non-Asian), and prior laxative/enema use. The subgroup analysis of the male patient data was based on a relatively small sample size (approximately 10% of the patients), so interpretation should be made with caution. Further research to confirm the efficacy of prucalopride in male patients with chronic constipation is currently on-going in Europe. Once-daily administration of prucalopride showed a rapid onset of action and the treatment effects were maintained throughout the treatment period. The therapeutic benefit of prucalopride was also demonstrated by an average increase of ≥1 SCBM per week, reduced use of rescue laxative/enema, improved constipation-related bowel symptoms, and enhanced quality of life.

Prucalopride was safe and well tolerated in patients in the Asia-Pacific region, and the study did not reveal any unexpected safety findings among treatment-emergent adverse events, laboratory values, vital sign measurements, or ECG recordings. Most adverse events were reported as mild or moderate in severity and were transient, and assessed by the investigators as not related to study drug. A low percentage of patients had adverse events that led to study discontinuation. The findings were generally similar in the placebo and prucalopride groups for all hematology and blood chemistry analytes, pulse rate and blood pressure, and ECG parameters. Prucalopride treatment was not associated with QTc prolongation. One patient without cardiovascular disease history was diagnosed with heart ischemia on ECG with no symptoms from the protocol-specified ECG at Day 28. The patient was seen by a cardiologist who indicated that the ECG change was only a non-specific T-wave abnormality but not significant, and did not require treatment. The investigator assessed this event as mild in intensity and possibly related to study drug.

This study was modeled after three identical double-blind, placebo-controlled pivotal studies that enrolled patients with CC predominantly from Western populations and treated them with prucalopride at daily doses of 2 or 4 mg for 12 weeks.^18–20^ The primary results of these three pivotal studies have been summarized in review articles of prucalopride as a treatment for constipation.^10^^,^^13^

Across the three pivotal studies, treatment with prucalopride 2 or 4 mg resulted in a significantly higher proportion of patients with normalization of BMs compared with placebo. Over the 12-week treatment period, 23.6% of patients receiving prucalopride 2 mg achieved ≥3 SCBMs per week compared to 11.3% of placebo patients (pooled population). The beneficial effect of treatment was evident over the first 4 weeks and was maintained over the 12 weeks of the study. All three pivotal studies showed a statistically significant and consistent effect on a wide range of secondary endpoints that assess clinically relevant aspects of CC. The 2-mg dose provided comparable efficacy to the 4-mg dose for all efficacy parameters. Both 2 and 4 mg prucalopride were safe and well tolerated with no unexpected safety findings among treatment-emergent adverse events, laboratory values, vital sign measurements, or ECG recordings.

In summary, the overall efficacy and safety of prucalopride 2 mg as a treatment for CC appear similar for patients from the Asia-Pacific region and from Western populations.^10^^,^^16^^,^^30^ Ethnicity shows the potential to affect a drug’s efficacy and safety profile, possibly as a result of pharmacokinetic and pharmacologic variations,^25^^,^^26^ this does not appear to be the case with prucalopride treatment for constipation.^16^

The aforementioned studies of prucalopride evaluated treatment for CC over 12 weeks. Patients who completed the three pivotal studies were invited to continue prucalopride treatment in two open-label, long-term, follow-up studies with similar design, and the pooled results show that the improvements in patient satisfaction with bowel movements and treatment, as observed after 4 and 12 weeks of double-blind treatment, was maintained for at least 18 months.^21^ Based on an experience of 1464 patient-years exposure to prucalopride, 41–50% of patients did not need laxatives in addition to prucalopride treatment to maintain satisfactory control of CC. Future studies may be beneficial to further evaluate the long-term efficacy and safety of prucalopride.

The patients enrolled in this study had chronic disease and were dissatisfied with their previous treatment. Approximately 60% of patients had a history of constipation for greater than 10 years, and over half of the patients reported no adequate relief of their constipation with the use of laxatives and enemas. The majority of patients were women (90%), which is indicative of the higher prevalence of CC in women and the fact that women are more likely to consult their physician.^3^^,^^9^ The therapeutic benefit of prucalopride as a prokinetic for constipation treatment occurred in women and men and other subgroups (e.g., age, race, prior laxative/enema).

## Conclusion

In patients in the Asia-Pacific region with CC, prucalopride 2 mg given once-daily significantly improved bowel function, associated symptoms, and satisfaction in CC over a 12-week treatment period. Prucalopride was safe and well tolerated by patients in the Asia-Pacific region.

## Author Contribution

MK contributed to the conception and design of the research and assisted in protocol development; all authors were involved in the acquisition of data or analysis and interpretation of data, and were involved in the initial drafting of the manuscript. All authors critically reviewed and contributed to subsequent drafts and approved the submission. The authors had complete access to the data that support the publication.

## Supporting Information

Data S1 Treatment-emergent abnormalities in electrocardiogram parameters.
