# Supplementary material for: Prucalopride in the treatment of chronic constipation in patients from the Asia-Pacific region: a randomized, double-blind, placebo-controlled study
Source: Neurogastroenterol Motil. 2012 Oct 11;24(11):999–e541. doi: 10.1111/j.1365-2982.2012.01983.x (PMC3509366; doi:10.1111/j.1365-2982.2012.01983.x)
Supplement: Data S1 — Treatment-emergent abnormalities in electrocardiogram parameters. [file nmo0024-0999-SD1.doc]

| Treatment-emergent abnormalities in electrocardiogram parameters | | | | |
| --- | --- | --- | --- | --- |
|  | **Placebo** | | **Prucalopride** | |
| **ECG parameter abnormality, n (%)** | **N** | **n (%)** | **N** | **n (%)** |
| Heart rate |  |  |  |  |
| Low | 238 | 4 (1.7) | 242 | 0 (0.0) |
| High | 238 | 0 (0.0) | 242 | 0 (0.0) |
| PR interval |  |  |  |  |
| High | 238 | 3 (1.3) | 230 | 0 (0.0) |
| QRS width |  |  |  |  |
| Narrow | 244 | 0 (0.0) | 243 | 0 (0.0) |
| Wide | 244 | 0 (0.0) | 243 | 0 (0.0) |
| QT interval |  |  |  |  |
| High | 242 | 0 (0.0) | 240 | 0 (0.0) |
| QTcF classification, prolonged |  |  |  |  |
| Week 4 LOCF | 241 | 0 (0.0) | 234 | 2 (0.9) |
| Week 12 LOCF | 247 | 0 (0.0) | 244 | 0 (0.0) |
| QTcB classification, prolonged |  |  |  |  |
| Week 4 LOCF | 241 | 3 (1.2) | 234 | 4 (1.7) |
| Week 12 LOCF | 247 | 3 (1.2) | 244 | 3 (1.2) |
| N, patients with normal baseline value; n, patients with abnormal post-baseline value. Heart rate: low: ≤50 beats/minute, high: ≥120 beats/minute; PR interval: high: ≥210 ms; QRS width: narrow: ≤50 ms, wide: ≥120 ms; QT interval: High: ≥500 ms; QTcF and QTcB intervals: prolonged >450 ms (male) or >470 ms (female). LOCF, last observation carried forward. | | | | |

**Supporting information**
